# Patterns of microbial contamination on Northumberland Strait shores

**DOI:** 10.1371/journal.pone.0315742

**Published:** 2025-01-30

**Authors:** Miranda E. Corkum, Naaman M. Omar, Douglas A. Campbell

**Affiliations:** Department of Biology, Mount Allison University, Sackville, NB, Canada; DIU: Dhaka International University, BANGLADESH

## Abstract

The re-emergence of episodic faecal contamination of Parlee and Murray Corner beaches, on the Northumberland Strait of New Brunswick, Canada, in 2017, raised renewed community concerns on the health, environmental and tourism sustainability of these community resources, and led to creation of an Integrated Watershed Management Plan for the Shediac Bay Watershed (October 2021). In response we have to date compiled, curated and made accessible 205,772 microbial water quality data records spanning over 80 years from Southeastern New Brunswick and the Northumberland Strait. This dataset derives in large part from Shellfish Surveys completed by Environment and Climate Change Canada, along with data generated by multiple government agencies, Non-Governmental Organizations and citizen science sources. Records derived from these multiple sources are now deposited in the Gordon Foundation’s DataStream (https://atlanticdatastream.ca), an open access common platform for sharing structured information on fresh and marine water health, delivered on a pan-Canadian scale, in collaboration with regional monitoring networks. We herein outline our data assembly, curation and deposition, along with preliminary analyses of contamination patterns at three representative sites on the Northumberland Strait coast of New Brunswick. Our results suggest that cumulative rainfall over 48 h is useful in predicting contamination risk at the developed Parlee Beach, and thereby demonstrate how open data can be used to inform policy and management decisions.

## Introduction

The Northumberland Strait off New Brunswick, Canada, is shallow (65 m maximum depth), and characterized by shorelines with low elevation gradients, extensive coastal dunes, and multiple bays and estuary systems, which make it especially vulnerable to effects of climate change [1, 2]. The coast has a long history of human use for shellfish harvesting, coastal fisheries, farming, intense recreational use during the warm summers, and now increasing residential development linked to steady population growth [3]. Preserving high water quality in the Northumberland Strait is therefore crucial for the well-being of its marine ecosystems, shellfish harvesting and tourism industries, which support the local culture and economy. *Enterococcus*, *Escherichia coli* (*E*. *coli*) and Faecal Coliforms found in water are widely used as readily detectable faecal indicator bacteria, predicting the potential presence of other pathogenic microorganisms of faecal origin [4]. As such, they are used to monitor, assess and predict microbial water quality, to manage and regulate food harvesting [5] and recreational activities [6, 7].

Faecal contamination can enter water bodies as a result of heavy rainfall and stream discharge when overland runoff from precipitation carries faecal indicator bacteria from agricultural lands, pet excrement (on or adjacent to beaches, or drainage courses) or lift station overflows [8–11]. Sub-standard septic systems or leaking sewage connections, boat sewage discharges on the water or from local marinas, shorebirds and bather load at public beaches can also contribute to faecal contamination [12–14]. Beyond direct contamination, *in situ* growth of indicator faecal indicator bacteria is positively associated with increased air and water temperatures [15], which can influence or distort interpretations of origins of contamination. Nevertheless, Faecal Indicator Bacteria are widely used inputs to models for managers in making shellfish harvesting, beach posting and closure decisions [16, 17].

Repeated detections of faecal indicator bacteria at Parlee Beach (lat 46.239706°, long -64.509637°), on the Northumberland Strait shore of New Brunswick, raised community concerns in 2016–2017, leading to an Integrated Watershed Management Plan for the Shediac Bay Watershed, on the Northumberland Strait [18]. In response we assembled microbial water quality data records for the Northumberland Strait, derived largely from many years of Shellfish Surveys completed by Environment and Climate Change Canada [19], along with data records from provincial and municipal government agencies, Non-Governmental Organizations and citizen science sources. These data on faecal contamination of coastal and estuarine waters are now deposited in the DataStream open access database [20], sponsored by the Gordon Foundation. DataStream does not require water quality samples to have been analyzed in a certified lab in order to be published on their site. However, in times of increasing environmental stress due to climate change, development, and budgetary restraints within governmental environmental monitoring programs, Community Based Water Monitoring is an increasingly important aspect of water governance and usage decisions [21]. Platforms like DataStream provide support and guidance to the water stewards who monitor ecosystem health, by ensuring standardized accessibility of data and meta-data. DataStream encourages groups who deposit data to provide as much information on the sample collection, laboratory analysis, and certifications (if any) as possible, so users make informed decisions on whether given data points or sources are useful for a particular purpose.

**Fig 1:**
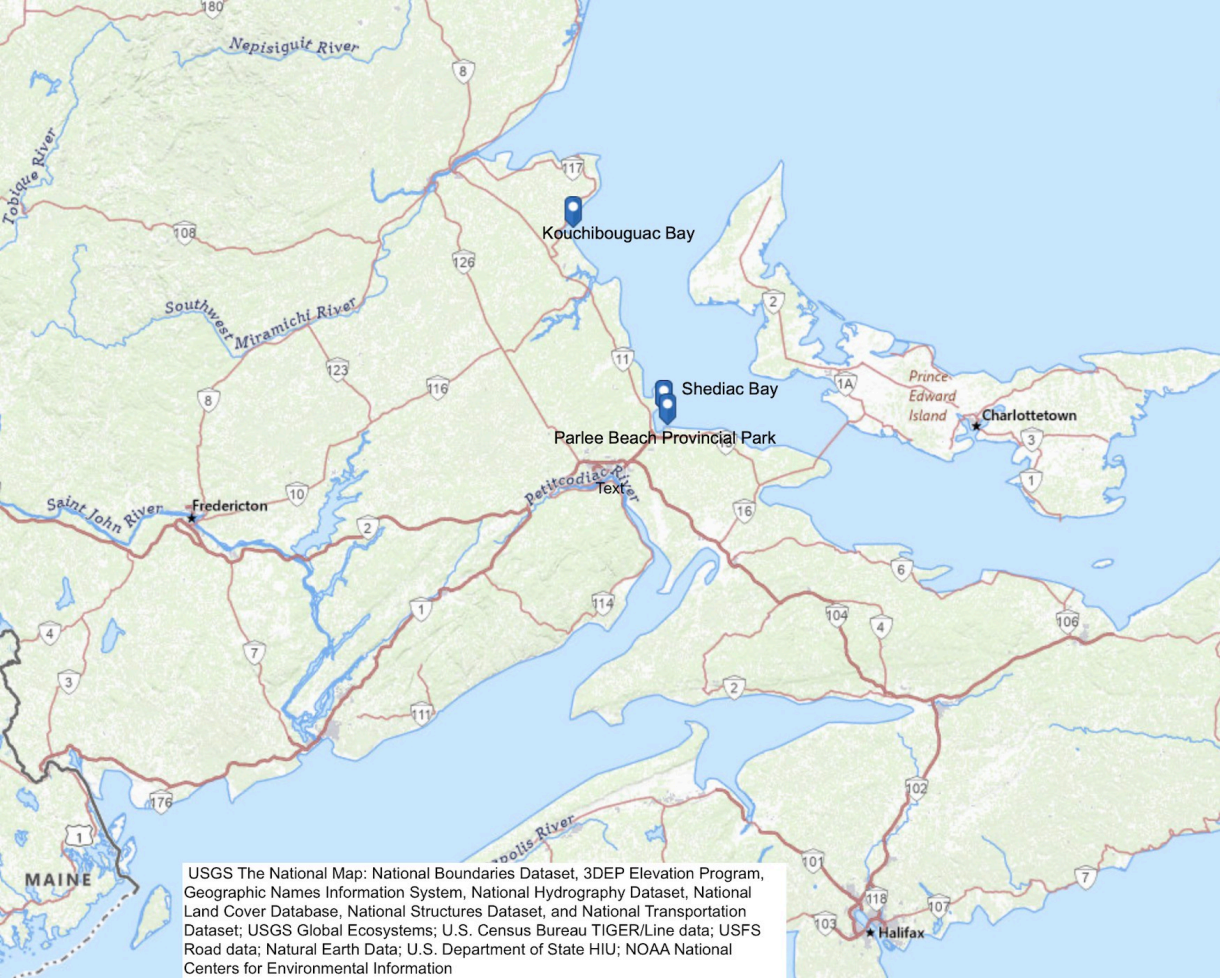
Study sites—Kouchibouguac Bay, Shediac Bay and Parlee Beach Provincial Park, Northumberland Strait, New Brunswick, Canada. Mapped using the open access USGS National Map Viewer (https://www.usgs.gov/tools/national-map-viewer).

We herein outline our data assembly, curation and deposition, along with preliminary analyses of contamination patterns at three representative sites on the Northumberland Strait coast of New Brunswick (Fig 1), over years, seasons, and in response to weather, to demonstrate how open data can be used to inform policy and management decisions to improve public health outcomes.

### Study sites

**Kouchibouguac Bay** (lat 46.848534°, long -64.929960°) is a shallow bay located in a less developed, predominantly forested area within Kouchibouguac National Park. Although a popular summer tourist destination, the park is closely regulated and restricted to recreational activities. It received 243,489 visitors in 2022–2023 [22]. Temperature and rainfall data for Kouchibouguac Bay were taken from Environment and Climate Change Canada station “KOUCHIBOUGUAC”: Station ID 26968 (lat 46.78°, long -65.02°) for years 2000–2015 and Station ID 54538 (lat 46.79°, long -65.01°) for years 2016–2022.

**Shediac Bay** (lat 46.281516°, long -64.524428°) is a shallow bay adjacent to the town of Shediac (population ~8000 [3]) and other smaller villages, with Parlee Beach situated at the southern entrance to the bay. Shediac Bay has a long history of development with a mixture of commercial activity, fisheries industry, large residential developments, cottages, marinas, docks, small farms and single residences close to shore. Temperature and rainfall data for Shediac Bay were taken from Environment and Climate Change Canada station “MONCTON/GREATER MONCTON ROMEO LEBLANC INTL A”: Station ID 6207 (lat 46.79°, long -65.01°) for years 2010–2011 and Station ID 50309 (lat 46.79°, long -65.01°) for years 2012 to 2023.

**Parlee Beach Provincial Park** (lat 46.239706°, long -64.509637°), at the mouth of Shediac Bay, is one of the most popular beaches in Eastern Canada with 736,426 visitors in 2021–2022 [23]. Parlee Beach is immediately adjacent to dense cottage development in Pointe-du-Chêne, New Brunswick, and hosts a restaurant on the park beach itself. Temperature and rainfall data for Parlee Beach were taken from Environment and Climate Change Canada station “MONCTON/GREATER MONCTON ROMEO LEBLANC INTL A”: Station ID 6207 (lat 46.79, long -65.01) for years 2010–2011 and Station ID 50309 (lat 46.79°, long -65.01°) for years 2012 to 2023.

## Materials and methods

Microbial water quality data records exist dating back over 80 years for Southeastern New Brunswick and the Northumberland Strait. Records, totalling 205,772, derive in large part from Shellfish Surveys completed by Environment and Climate Change Canada [19], along with data generated by various government agencies, Non-Governmental Organization and citizen science sources. Records were obtained via access to information requests submitted by concerned citizens in the Shediac Bay area, our internet, archive, and library searches of government reports, and from direct communication with Environment and Climate Change Canada staff. Data from these diverse sources required extensive tidying and curation by Mount Allison University undergraduate students and research staff, for assembly into a standardized format suitable for deposit [24] into the Gordon Foundation’s DataStream (https://atlanticdatastream.ca), an open access common platform for sharing structured information on fresh and marine water health delivered on a national scale, in collaboration with regional monitoring networks.

In response to public demand for transparency in water quality reporting and resulting media coverage, New Brunswick Department of Health now posts bacteria contamination data for various public beaches, including intensive sampling through the summer months for Parlee Beach Provincial Park [25]. The Environment and Climate Change Canada Shellfish Water Classification Program data is now publicly accessible through Open Canada [19], and these records are being uploaded to DataStream, separately from our assembly and curation efforts for data from the Northumberland Strait shores of New Brunswick.

A sub-set of 7173 records from the curated Northumberland Strait dataset, from years 2010–2023, for Kouchibouguac Bay, Shediac Bay and Parlee Beach, was analyzed in this paper. The two main data sources are Environment and Climate Change Canada Shellfish Survey (Kouchibouguac and Shediac Bay–samples analyzed at an in-house laboratory) and New Brunswick Department of Health (Parlee Beach Provincial Park–samples analyzed at Research and Productivity Council certified laboratories). While *Enterococci* are now considered the best available indicator of water quality for marine recreational waters [26–28], current monitoring programs in Canada still routinely use Faecal Coliforms or *Escherichia coli*. The Environment Canada and related surveys for shellfish harvesting continue to use Faecal Coliforms, and provide a deep temporal record across many sites. Therefore, in spite of the advantages of *Enterococcus* for marine water quality sampling, we focus on Faecal Coliforms and *E*. *coli*.

Bacterial counts were reported as “Faecal Coliform” or as “*Escherichia coli*”, with units of Most Probable Number (MPN) or Colony Forming Units (CFU) per 100 mL. Bacterial counts were in most cases used as reported, however, values recorded as above or below a limit of analytical detection were handled as follows. To cope with plotting on log_10_ scale, bacterial counts recorded as zero CFU/100 mL (i.e. Parlee Beach year 2017) are here plotted and analyzed as “1 CFU/100 mL”. Bacterial counts recorded as falling below a limit of detection of two CFU/100 mL (“< 2”), are here plotted and analyzed as “1 CFU/100 mL”. Bacterial counts recorded as falling below a limit of detection of ten MPN/100 mL (“< 10”), are here plotted and analyzed as “5 MPN/100 mL”. Bacterial counts recorded as falling above a limit of detection of 400 CFU/100 mL (“> 400”), (i.e. Parlee Beach, August 8, 2021) are here plotted and recorded as “400 CFU/100 mL”.

Daily precipitation and air temperature data for the three sites were accessed from Environment and Climate Change Canada [29] using the ‘weathercan’ [30] package. Assembly of bacterial count data, weather data, analysis and presentation were done using the ‘knitr’ [31], ‘tidyverse’ [32], ‘datastreamr’ [33], ‘lutz’ [34], ‘kableExtra’ [35], ‘viridis’ [36], ‘stats’ [37] and ‘broom’ [38] packages, running under R [39] and R Studio [40]. Bacterial counts were plotted, analyzed and compared to matched sampling date, total cumulative precipitation, and mean air temperature over 48 h prior to sample collection date for years 2010 to 2023. For most bacterial counts we do not have time of day for sampling, so we could not pursue finer scale temporal analyses.

Health Canada epidemiological guidelines for swimming used in this analysis [6, 41] are a) A geometric mean of most recent five samples ≤ 200 *E*. *coli* per 100 mL OR b) a single-sample maximum ≤ 400 *E*. *coli* per 100 mL. Note that Health Canada guidelines updated in 2023 have lowered the maximum recommended single sample *E*. *coli* value from ≤ 400 CFU per 100 mL to < 235 CFU per 100 mL [7]. Environment and Climate Change Canada Shellfish Survey approves shellfish harvest areas if a) the median or geometric mean faecal coliform MPN of the water is ≤ 14 per 100 mL AND b) not more than 10% of the samples exceed a faecal coliform MPN of 43 per 100 mL, for a five-tube decimal dilution test [4]. For plotting and analyses we use reference lines of 200 and 43 bacterial counts per 100 mL.

The temporal and geographic resolution of Environment Canada precipitation data reporting varied across the study sites. We therefore used only cumulative precipitation in our model as attempting to extract maximum rates of precipitation would broaden approximations which would in turn lower the statistical power of any analyses.

Other potentially accessible parameters, not considered in the presented analyses but which might influence patterns of microbial contamination, include maximum shorter term rates of precipitation, tide, solar irradiation, water temperature, significant wave height, offshore wind speed, and spatial and temporal analyses of potential point sources of contamination including industry, agriculture and sewage facilities.

## Results

**Fig 2:**
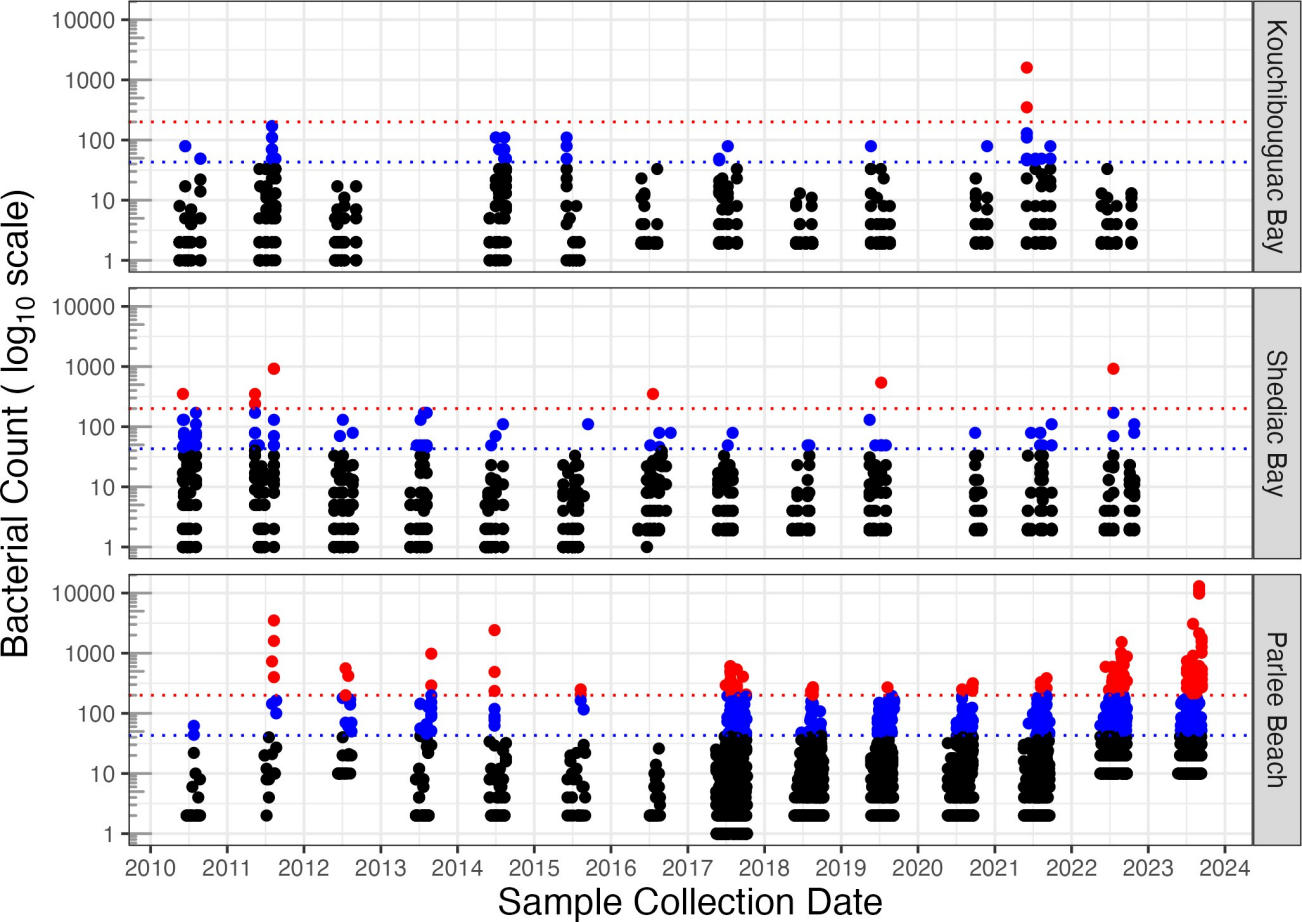
Bacterial count vs. sample collection date at study sites. Faecal Coliform or Escherichia coli per 100 mL (log_10_ scale) from years 2010 to 2023, with thresholds of 200 (red dotted line) or 43 (blue dotted line) bacteria per 100 mL. Parlee Beach had increased sampling intensity from year 2017 onward.

For years 2010–2023 we firstly plot bacterial count (Faecal Coliform or *Escherichia coli* per 100 mL) vs. sample collection date (Fig 2).

In subsequent statistical analyses sampling date is included as a co-variant, to capture any temporal trends in contamination events.

**Fig 3:**
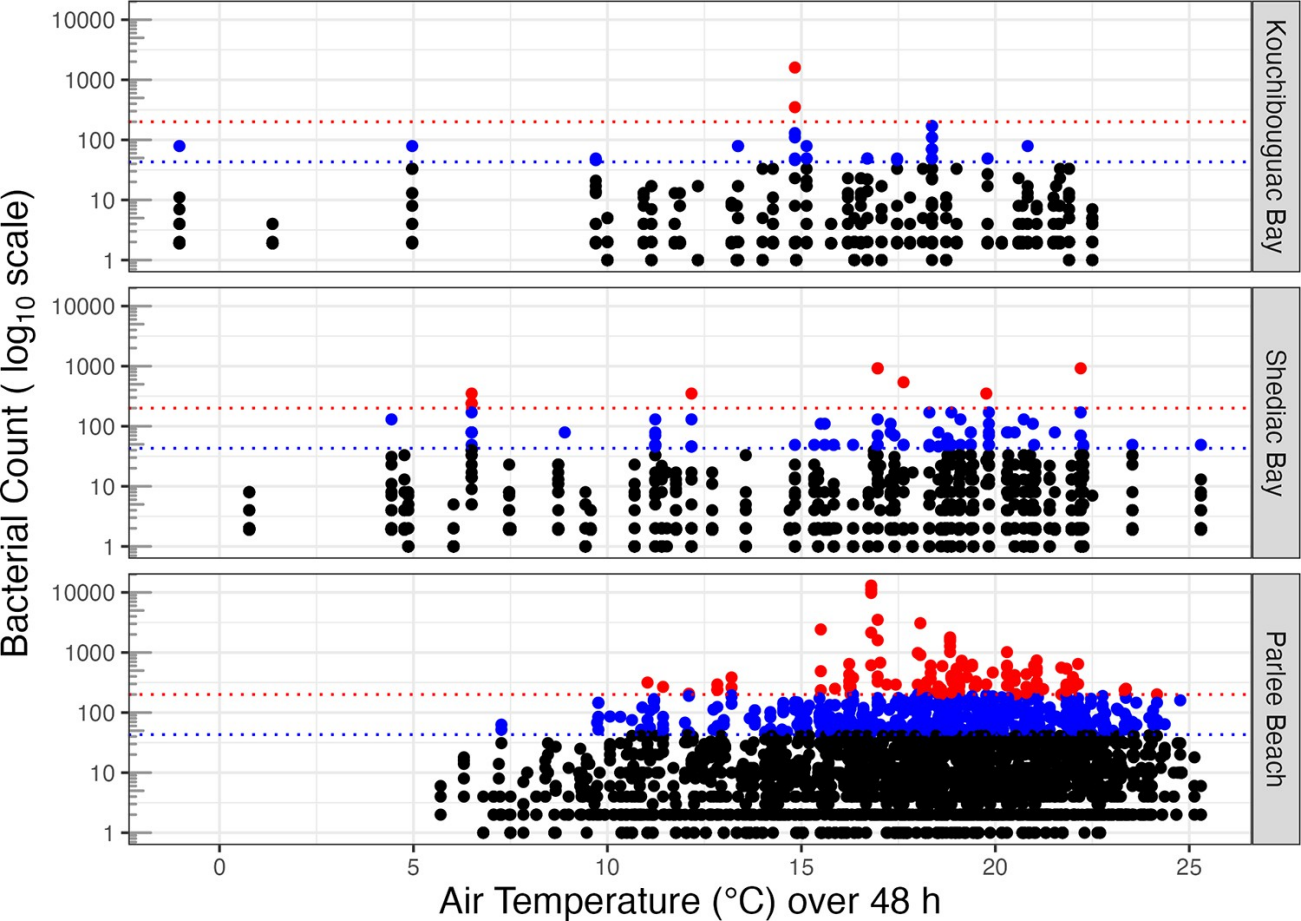
Bacterial count vs. average air temperature (°C) over the preceding 48 h at study sites. Faecal Coliform or Escherichia coli per 100 mL (log_10_ scale) from years 2010 to 2023, with thresholds of 200 (red dotted line) or 43 (blue dotted line) bacteria per 100 mL.

We next plotted bacterial counts vs. average air temperature (°C) over the preceding 48 h, from the weather stations nearest the respective sampling sites (Fig 3).

Temperature at the time of sampling at all three study sites showed a left-skewed distribution [42] peaking at around 18°C (S1 Fig.).

**Fig 4:**
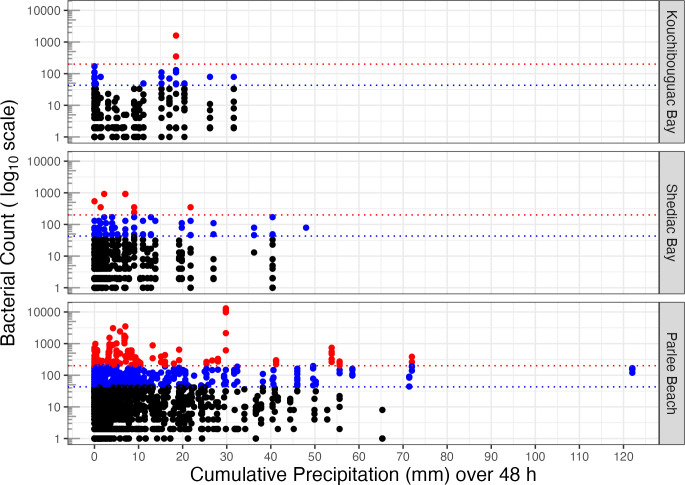
Bacterial count vs. cumulative precipitation (mm) over preceding 48 h at study sites. Faecal Coliform or Escherichia coli per 100 mL (log_10_ scale) from years 2010 to 2023, with thresholds of 200 (red dotted line) or 43 (blue dotted line) bacteria per 100 mL.

We next plotted bacterial counts vs. cumulative precipitation (mm) over the preceding 48 h (Fig 4) from the weather stations, since coastal contamination often results from run off [43]. Note that most measurement points fell on days with low cumulative precipitation over the preceding 48 h, a pattern partially obscured by heavy overlap of many points below ~ 10 mm cumulative precipitation.

**Fig 5:**
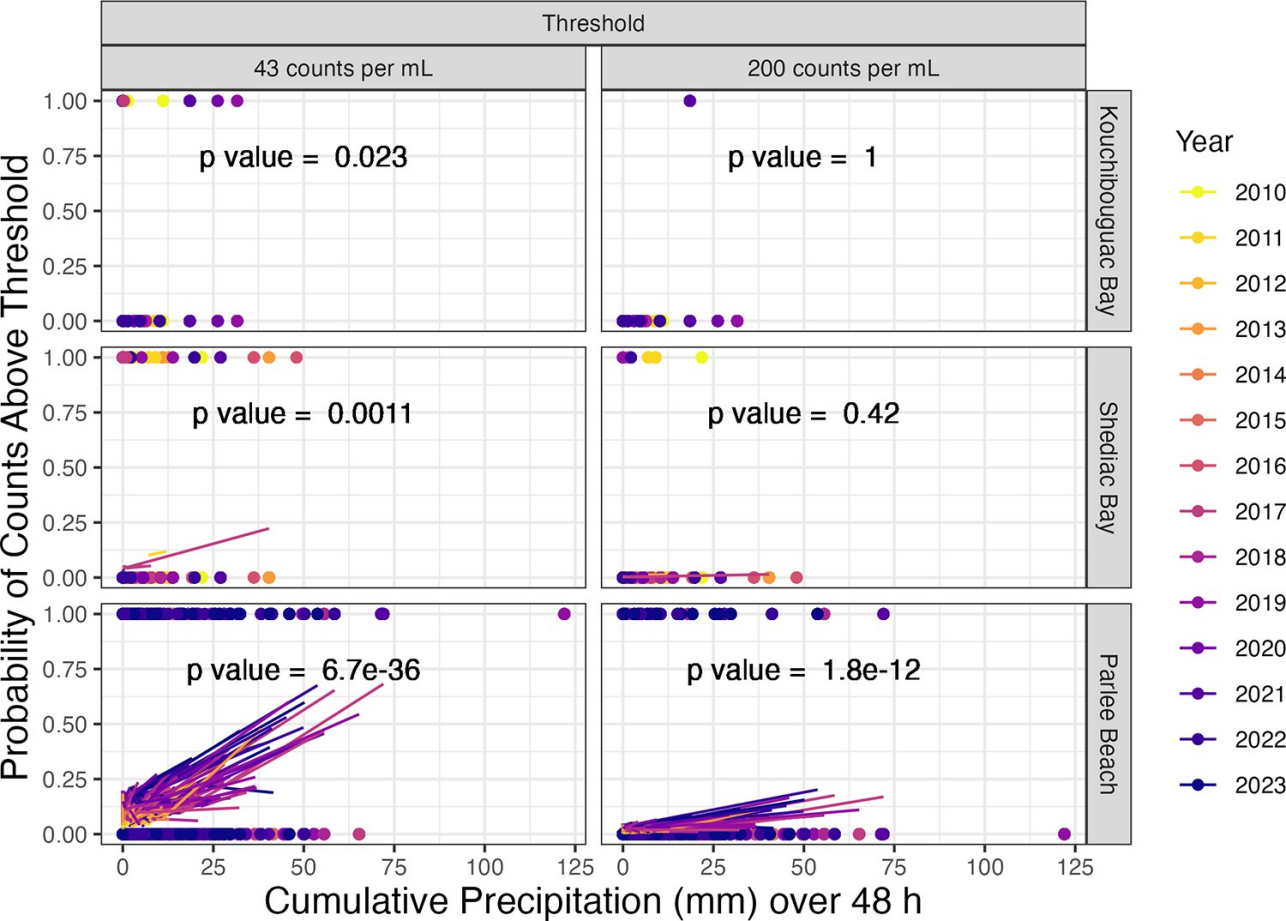
Binomial probabilities of bacterial count above thresholds, vs. cumulative precipitation (mm, 48 h). Sample year (2010 to 2023) and mean temperature (°C) over preceding 48 h are co-variants. See Table 1 for model slopes and p values.

To uncover potential predictors of contamination risk, we used a binomial analyses of the probability of bacterial counts falling above regulatory thresholds, in response to cumulative precipitation over 48 h (Fig 5, Table 1).

**Table 1:** Binomial probabilities and p values for bacterial count above thresholds, vs. cumulative precipitation (mm, 48 h). Sample year (2010 to 2023) and mean temperature (°C) over preceding 48 h are covariates.

Location	Bacterial Threshold	Precip 48 h	Year	Temp 48 h	p, Precip 48 h	p, Year	p, Temp 48 h
Kouchibouguac Bay	43	0.06	0.05	0.05	0.02	0.23	0.19
Shediac Bay	43	0.04	-0.12	0.00	0.00	0.00	0.95
Parlee Beach	43	0.04	0.14	0.06	0.00	0.00	0.00
Kouchibouguac Bay	200	1.90	2.11	3.08	1.00	1.00	1.00
Shediac Bay	200	0.03	-0.17	-0.09	0.42	0.11	0.15
Parlee Beach	200	0.03	0.15	0.07	0.00	0.00	0.00

At Kouchibouguac Bay (Fig 5), precipitation over the preceding 48 h had a significant influence on the probability of bacterial counts falling above 43 bacteria per 100 mL threshold (Table 1). Neither successive sampling years nor temperature over preceding 48 h, showed a significant influence on the probability of counts above the 43 bacteria per 100 mL threshold. At Kouchibouguac Bay we found only one data point falling above the 200 bacteria per 100 mL contamination threshold (Fig 5), and so precipitation over the preceding 48 h, nor sampling year, nor temperature show a significant influence on the probability of bacterial counts falling above 200 bacteria per 100 mL threshold (Table 1).

At Shediac Bay (Fig 5), precipitation over the preceding 48 h had a significant influence on the probability of bacterial counts falling above 43 bacteria per 100 mL threshold, while each successive year decreased the probability of counts above the 43 threshold (Table 1). Neither precipitation over the preceding 48 h, nor sampling year, nor temperature show a significant influence on the probability of bacterial counts falling above 200 bacteria per 100 mL threshold (Table 1).

At Parlee Beach (Fig 5), precipitation over the preceding 48 h, successive sampling year and temperature over the preceding 48 h all had a significant influence on the probability of bacterial counts falling above both the 43 and 200 bacteria per 100 mL thresholds (Table 1). A comparative model run with bacterial count above 200 bacteria per 100 mL threshold, vs. cumulative precipitation (mm) over 48 h, with heaviest rain days removed (> 40 mm cumulative precipitation over 48 h), showed similar results (S1 Table).

## Discussion

At Parlee Beach Provincial Park bacterial counts falling above the advisory threshold of 200 bacteria per 100 mL trended downwards up until 2020, possibly as a result of management and policy interventions [18, 44–48]. In more recent years days above the 200 bacteria per 100 mL threshold are again increasing.

The lack of significant influence of temperature on bacterial counts falling above thresholds at Kouchibouguac and Shediac Bay, suggests faecal bacteria are not growing in the water at these beaches during warmer months, but that episodes of bacterial counts above threshold rather reflect bacteria washed into the beach water. In contrast, temperature does show a significant influence on bacterial counts falling above thresholds at Parlee Beach, so that lagged 48 h temperature may be a useful element in predicting contamination risk. Future analysis could untangle whether temperature has a direct effect on contamination risk or whether temperature is a co-variant of factors such as visitor numbers which could drive increases in bacterial counts.

We interpret the lack of influence of lagged 48 h precipitation on faecal indicator bacteria at Kouchibouguac Bay as a reflection of the relatively protected shorescape in the Kouchibouguac National Park, acting to buffer the coastline from pulses of terrestrially derived contamination through run-off. Shediac Bay is a more developed shorescape, but relatively large water flows in the estuary, and multiple diffuse sources may dampen any statistical detection of faecal indicator bacteria responses to lagged 48 h precipitation.

Precipitation summed over 48 h shows a significant influence on the probability of bacterial counts falling above the advisory threshold of 200 bacteria per 100 mL at Parlee Beach, suggesting local overland runoff or sewage lift station overflows as contamination sources, since summed 48 h precipitation does not influence the probability of bacterial counts above the advisory threshold of 200 bacteria per 100 mL at the less developed Kouchibouguac Bay nor in the wider Shediac Bay. The influence of 48 h precipitation is significant, but by no means absolute, since over many periods of high precipitation bacterial counts remained below the threshold. Similar studies in the Toronto region of Ontario, Canada [15] and in Vancouver, Canada [17] found increased total rainfall in the preceding 48 h was also positively associated with increased *E*. *coli* concentrations.

Lagged 24 h rainfall has not been a reliable predictor of water quality at Parlee Beach [25]. We suggest that lagged 48 h rainfall may instead be a useful predictor of contamination risk at Parlee Beach, and may give temporal clues as to the sources of contamination, which appear to take more than 24 h to influence bacterial counts in the water at the beach. This is of particular concern considering the expected increase in frequency and intensity of extreme rainfall events due to climate change [49].

Future data analyses of Northumberland’s coastal beaches could consider the interacting effects of tide and high wind events on water quality, as intertidal sediment can be a reservoir for bacteria, notably *Enterococcus* [50, 51]. Access to local anemometer data for wind monitoring would support these analysis.

Water testing is logistically complex, expensive and involves significant turnaround times (i.e. 24 hours) between water sample collection, potential decisions and reporting. This makes beach closure orders much less effective as they often miss bacteria spikes. Predictive modeling of risks of microbial contamination can help focus testing efforts and even allow pre-emptive warnings of potential contamination, faster than is feasible with standard culture-based testing methods.

Data collected by diverse New Brunswick watershed groups, municipal, provincial and federal government can be assembled and integrated with other data sources to help make strategic regional-based decisions to protect our water and recreational resources.

## Supporting information

S1 Fig Bacterial counts per 100 mL show a left-skewed distribution vs. air temperature (°C). P values for departure from normality were determined using the Shapiro-Wilk test.
(ZIP)

S1 Table Binomial probabilities and p values for bacterial count above thresholds, vs. cumulative precipitation (mm, 48 h). Heaviest rain days (more than 40 mm cumulative precipitation over 48 h) were removed from analysis. Sample year (2010 to 2023) and mean temperature (°C) over preceding 48 h are co-variants.
(PDF)

S1 Appendix Data dictionary. (CSV)
