# Supplementary figures and images for: Patterns of microbial contamination on Northumberland Strait shores
Source: PLoS One. 2025 Jan 30;20(1):e0315742. doi: 10.1371/journal.pone.0315742 (PMC11781611; doi:10.1371/journal.pone.0315742)

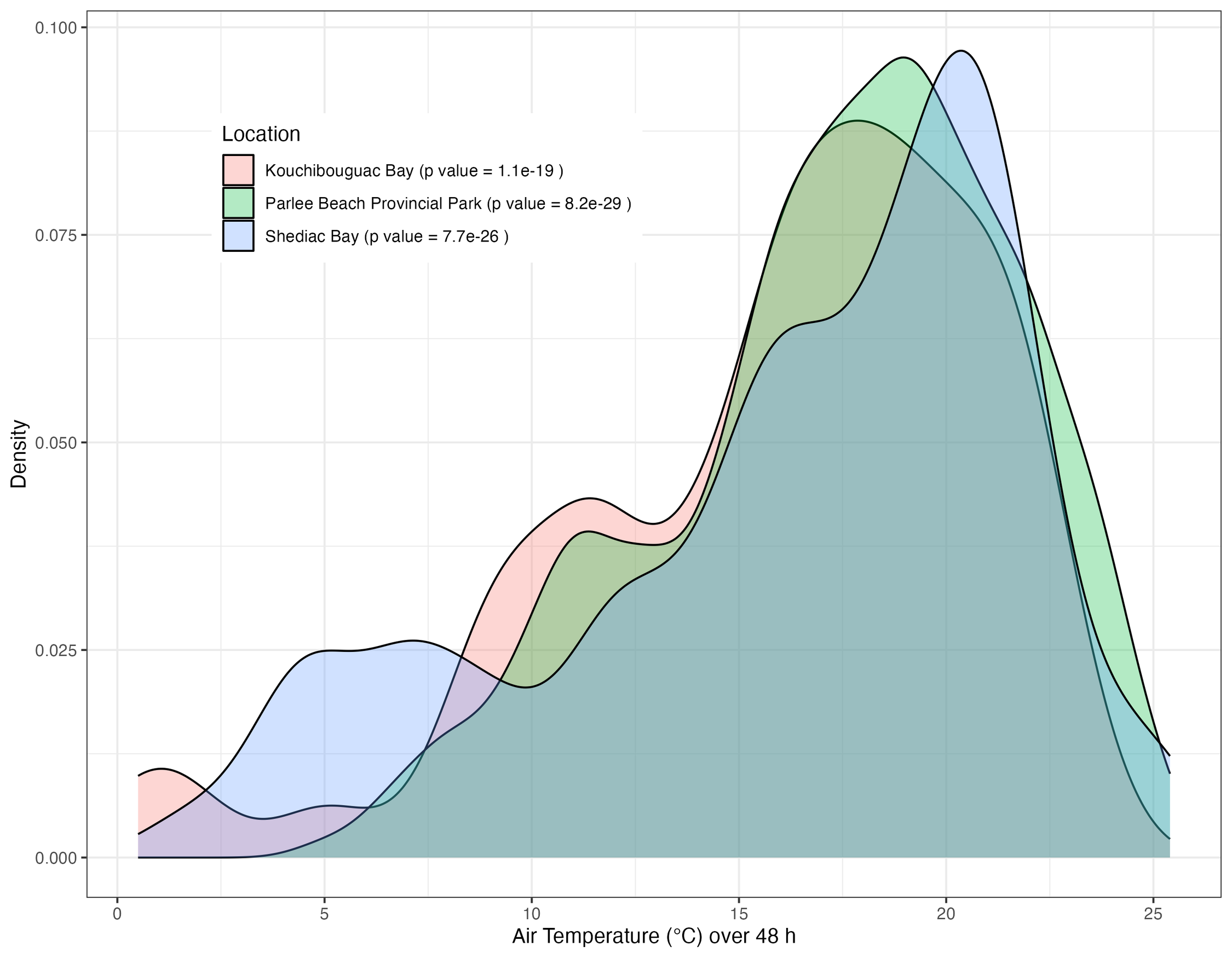

Supplement: S1 Fig — P values for departure from normality were determined using the Shapiro-Wilk test. (ZIP) [file pone.0315742.s001.zip › S1_Fig.tif]
