# Supplementary material for: Patterns of microbial contamination on Northumberland Strait shores
Source: PLoS One. 2025 Jan 30;20(1):e0315742. doi: 10.1371/journal.pone.0315742 (PMC11781611; doi:10.1371/journal.pone.0315742)
Supplement: S1 Table — Heaviest rain days (more than 40 mm cumulative precipitation over 48 h) were removed from analysis. Sample year (2010 to 2023) and mean temperature (°C) over preceding 48 h are co-variants. (PDF) [file pone.0315742.s002.pdf]

Location	Precip 48 h	Year	Temp 48 h	p, Precip 48 h	p, Year	p, Temp 48 h
Kouchibouguac Bay	1.90	2.11	3.08	1.0	1.00	1.00
Shediac Bay	0.05	-0.16	-0.07	0.2	0.11	0.23
Parlee Beach	0.04	0.12	0.06	0.0	0.00	0.01
